# The Sulfate Supply Maximizing Arabidopsis Shoot Growth Is Higher under Long- than Short-Term Exposure to Cadmium

**DOI:** 10.3389/fpls.2017.00854

**Published:** 2017-05-22

**Authors:** Alessandro Ferri, Clarissa Lancilli, Moez Maghrebi, Giorgio Lucchini, Gian Attilio Sacchi, Fabio F. Nocito

**Affiliations:** ^1^Dipartimento di Scienze Agrarie e Ambientali – Produzione, Territorio, Agroenergia, Università degli Studi di MilanoMilano, Italy; ^2^Istituto d’Istruzione Superiore di CodognoCodogno, Italy

**Keywords:** *Arabidopsis thaliana*, cadmium, sulfate uptake, sulfate critical concentration, thiols

## Abstract

The processes involved in cadmium detoxification in plants deeply affect sulfate uptake and thiol homeostasis and generate increases in the plant nutritional request for sulfur. Here, we present an analysis of the dependence of Arabidopsis growth on the concentration of sulfate in the growing medium with the aim of providing evidence on how plants optimize growth at a given sulfate availability. Results revealed that short-term (72 h) exposure to a broad range of Cd concentrations (0.1, 1, and 10 μM) inhibited plant growth but did not produce any significant effects on the growth pattern of both shoots and roots in relation to the external sulfate. Conversely, long-term (22 days) exposure to 0.1 μM Cd significantly changed the pattern of fresh weight accumulation of the shoots in relation to the external sulfate, without affecting that of the roots, although their growth was severely inhibited by Cd. Moreover, under long-term exposure to Cd, increasing the sulfate external concentration up to the critical value progressively reduced the inhibitory effects exerted by Cd on shoot growth, indicating the existence of sulfate-dependent adaptive responses protecting the shoot tissues against Cd injury. Transcriptional induction of the high-affinity sulfate transporter genes (*SULTR1; 1* and *SULTR1; 2*) involved in sulfate uptake by roots was a common adaptive response to both short- and long-term exposure to Cd. Such a response was closely related to the total amount of non-protein thiols accumulated by a single plant under short-term exposure to Cd, but did not showed any clear relation with thiols under long-term exposure to Cd. In this last condition, Cd exposure did not change the level of non-protein thiols per plant and thus did not alter the nutritional need for sulfur. In conclusion, our results indicate that long term-exposure to Cd, although it induces sulfate uptake, decreases the capacity of the Arabidopsis roots to efficiently absorb the sulfate ions available in the growing medium making the adaptive response of *SULTR1; 1* and *SULTR1; 2* “*per se*” not enough to optimize the growth at sulfate external concentrations lower than the critical value.

## Introduction

Plants have evolved a complex network of adaptation mechanisms that allow them to minimize the damage from exposure to non-essential and potentially toxic metal ions (Clemens, 2001, 2006). Such mechanisms involve transport, chelation, and sequestration processes controlling metal homeostasis into the cells and throughout the whole plant.

The synthesis of cysteine (Cys)-rich metal binding peptides – such as phytochelatins (PCs) – appears as the most conserved and ubiquitous process used by plants for cadmium (Cd) detoxification. PCs are a class of small peptides consisting of repeating units of γ-glutamylcysteine (γ-Glu-Cys) followed by a C-terminal glycine (Gly): the general structure of these peptides is (γ-Glu-Cys)*_n_*-Gly, where *n* = 2 to 11 (Grill et al., 1987; Zenk, 1996; Cobbett, 2000; Cobbett and Goldsbrough, 2002), although PC2–PC4 are the most commonly detected PCs in both root and leaf tissues of Arabidopsis (Chen et al., 2006). The presence of γ-glutamyl linkages in these peptides implies that they are non-translationally synthesized using reduced glutathione (GSH) as the direct precursor in a transpeptidation reaction catalyzed by the enzyme PC synthase (Rea et al., 2004; Rea, 2006). Once synthetized in the cytoplasm, PCs form thiolate bonds with Cd^2+^ ions, and the resulting Cd-PC complexes are subsequently sequestered to the vacuole through multiple ABC-type transporters (Cobbett, 2000; Verbruggen et al., 2009; Mendoza-Cózatl et al., 2010; Park et al., 2012; Song et al., 2014; Brunetti et al., 2015).

In this way cells may control the concentration of the free Cd^2+^ ions in the cytosol, limiting the potential damage due to their overaccumulation. The relevance of this mechanism for the natural Cd tolerance of the plants has been underlined by the analysis of the Arabidopsis PC-deficient mutants *cad1* and *cad2-1*, which were more sensitive to Cd than wild-type plants (Howden et al., 1995a,b).

The interactions between Cd accumulation and sulfur (S) metabolism in higher plants have been exhaustively described and reviewed in several papers (Nocito et al., 2002, 2007; Mendoza-Cózatl et al., 2005; Ernst et al., 2008; Jozefczak et al., 2014; Lancilli et al., 2014; Khan et al., 2016). In particular, it has been shown that the increases in the metabolic request for both Cys and GSH – generated by Cd-induced PC biosynthesis – produce a demand-driven coordinated transcriptional regulation of genes involved in sulfate uptake, sulfate assimilation and GSH biosynthesis. Such an activation is thought to be pivotal for ensuring – at least in the early phases of Cd accumulation – both GSH homeostasis and adequate fluxes of reduced S to help Cd detoxification processes. In fact, the large amount of PCs produced following Cd exposure may generate additional sinks for thiols which, in turn, increase the total nutritional demand for S by plants. However, some aspects of this model need to be further elucidated, since it is not clear whether the early adaptive responses to Cd – involving the S assimilation pathway – are sufficient to maintain adequate S levels to promote plant growth under prolonged exposures to the metal, i.e., whether plants growing in the presence of Cd need a higher amount of S in the soil to maximize their growth. In fact the activation of GSH consuming activities due to Cd chelation may in turn reduce the GSH-related antioxidant capacity of the cells, generating further additional requests for thiols and, thus, for S. Considering these aspects, here we present and discuss two sets of experiments aimed at studying the effect of both short- and long-term exposure to Cd on the capability of Arabidopsis for optimizing growth under a wide range of sulfate concentrations in the growing medium, and showing that long-term exposure to Cd negatively affects this trait.

## Materials and Methods

### Plant Materials, Growth Conditions, and Experimental Design

*Arabidopsis thaliana* (Ler-0) seeds were washed under continuous shaking in 0.5 mL 0.01% (v/v) Tween 20 for 20 min, surfaced-sterilized by adding an equal volume of commercial bleach (4% active chlorine) for 5 min, and then rinsed four times with sterile distilled water. Seeds were sown on a sterile 3M^TM^ paper sheet – imbibed with sterile distilled water and laid down into a Petri dish – and then incubated for 4 days, in the dark, at 4°C to remove dormancy.

Vernalized seeds were sown, with a toothpick, on small pieces of rockwool (Grodan^®^) placed into appropriate seed holders, obtained by cutting 1 mL pipette tips at 2 and 12 mm from the tip. The seed holders were transferred into pipette tip boxes, filled with distilled water – to maintain the rockwool imbibed – and finally incubated at 22°C under continuous light to allow seed germination. Seven days after sowing, seedlings – selected for uniform growth – were transferred into 3 L plastic tanks (41 seedlings per tank) containing non-sterile aerated complete nutrient solutions and kept for 22 days in a growth chamber maintained at 22°C and 80% relative humidity, with a 12-h light period.

For the short-term Cd exposure experiments, plants were grown for 19 days in hydroponic solutions [250 μM NH_4_H_2_PO_4_, 1.5 mM KNO_3_, 1 mM Ca(NO_3_)_2_, 25 μM Fe-tartrate, 46 μM H_3_BO_3_, 9 μM MnCl_2_, 0.8 μM ZnCl_2_, 0.3 μM CuCl_2_, 0.1 μM (NH_4_)_6_Mo_7_O_24_, pH 6.5] with different sulfate concentrations (5, 25, 50, and 150 μM MgSO_4_). At the end of this pre-growing period plants were maintained in the same solutions and exposed, or not, to three concentrations of Cd^2+^ (0.1, 1, and 10 μM CdCl_2_) for 72 h.

For the long-term Cd exposure experiments plants were grown for 22 days in hydroponic solutions [250 μM NH_4_H_2_PO_4_, 1.5 mM KNO_3_, 1 mM Ca(NO_3_)_2_, 25 μM Fe-tartrate, 46 μM H_3_BO_3_, 9 μM MnCl_2_, 0.8 μM ZnCl_2_, 0.3 μM CuCl_2_, 0.1 μM (NH_4_)_6_Mo_7_O_24_, pH 6.5] under a wide range of sulfate concentrations (1, 2.5, 5, 7.5, 10, 25, 50, 75, 100, 125, and 150 μM MgSO_4_), in the presence or absence of 0.1 μM CdCl_2_.

In all the experiments, MgCl_2_ was added to maintain the same concentration (500 μM) of the Mg^2+^ ions in each solution. All hydroponic solutions were renewed daily to minimize sulfate depletion. At the end of the growing periods, plants were used for the *in vivo* experiments or harvested to be further analyzed. In this case, roots were washed for 10 min in an ice-cold 5 mM CaCl_2_ solution to displace extracellular Cd (Rauser, 1987), rinsed in distilled water and gently blotted with paper towels; shoots were separated from roots and the tissues were weighed before to being frozen in liquid N_2_ and stored at -80°C.

### Plant Growth Analysis

The curves describing the growth of both shoots and roots as a function of the sulfate concentration in the growing medium were drawn by fitting an exponential rise to maximum function to the data obtained by weighing the shoots and the roots of the Arabidopsis plants at the end of each experiment (Zhao et al., 1999). To this purpose, we chose a three-parameter exponential curve with the formula:

$$\begin{array}{c} y=y_{0}+a(1-e^{-bx}) \end{array}$$

where *y* is the fresh weight (FW), *y*_0_ is the FW offset from [SO_4_^2–^] = 0, *a* is the amplitude of the curve (i.e., the difference between the maximum and the minimum value of the FW), *b* is the rate constant, *x* is the sulfate concentration in the medium.

The sulfate critical concentration ([SO_4_^2–^]_crit_) – defined as the sulfate concentration in the growing medium that produced 95% of the maximum amount of fresh weight for shoots or roots – was calculated in each experiment as follows:

$$\begin{array}{c} {\left[{\mathrm{SO}}_{4}^{2-}\right]}_{\mathrm{crit}}=\frac{\log_{e}\left[\frac{0.05(y_{0}+a)}{a}\right]}{-b}. \end{array}$$

### Determination of Total Sulfur, Thiols, and Cadmium Content

Shoots and roots were pulverized using mortar and pestle in liquid N_2_. Total sulfur contents were determined according to the turbidimetric method described by Tabatabai and Bremner (1970). Total non-protein thiols (NPTs) and Cd contents were determined as described by Fontanili et al. (2016). Total GSH, reduced GSH and oxidized GSH (GSSG) were measured according to Griffith (1980). PCs and related peptides (non-GSH NTPs) were estimated as the difference between NPT and total GSH levels in both shoots and roots of Cd-exposed plants (Sghayar et al., 2015).

### Sulfate Influx Assay

Sulfate influx into the roots was measured by determining the rates of ^35^S uptake, over a 15 min pulse in a complete nutrient solution labeled with the radiotracer. Briefly, a single plant was placed onto 10 mL of a fresh acclimation nutrient solution with the same ionic composition as those used for plant growth, containing 150 μM MgSO_4_, supplemented or not with different concentrations of CdCl_2_ (0.1, 1, and 10 μM Cd^2+^ for the short-term exposure experiments; 0.1 μM Cd^2+^ for the long-term exposure experiments); each solution was maintained aerated and thermoregulated at 22°C. Radioactive pulses were started by adding ^35^S-labeled Na_2_SO_4_ to the uptake solutions. Specific activity was 4.7 kBq μmol^-1^. At the end of the pulse period, roots were rinsed twice for 1 min in 10 mL of a 4 mM CaSO_4_ non-radioactive solution at 4°C, blotted with paper towels, weighed, and then heated for 20 min at 80°C in 5 mL 0.1 N HNO_3_. Radioactivity was measured on aliquots of the extracting solution by liquid scintillation counting in a β counter (LS 6000SC, Beckman).

### RNA Extraction and qRT-PCR Analysis

Total RNA was extracted from roots using TRIzol^®^ Reagent (Life Technologies) and then purified using PureLink^®^ RNA Mini Kit (Life Technologies), according to the manufacturer’s instructions. Contaminant DNA was removed on-column using PureLink^®^ DNase (Life Technologies). First-strand cDNA synthesis was carried out using the SuperScript^TM^ III First-Strand Synthesis SuperMix for qRT-PCR (Invitrogen), according to the manufacturer’s instructions.

qRT-PCR analysis of *SULTR1; 1* (At4g08620) and *SULTR1; 2* (At1g78000) was performed on first-strand cDNA in a 20 μL reaction mixture containing GoTaq^®^ qPCR Master Mix (Promega) and the specific primers, using an ABI 7300 Real-Time PCR system (Applied Biosystems). The relative transcript level of each gene was calculated by the 2^-ΔΔCt^ method using the expression of the *S16* (At4g34620) gene as reference. Primers for qRT-PCR are listed in Supplementary Table S1.

### Statistical Analysis

Statistical analysis was carried out using SigmaPlot for Windows version 11.0 (Systat Software, Inc.). Quantitative values are presented as mean ± standard error of the mean (SE). Significance values were adjusted for multiple comparisons using the Bonferroni correction. Student’s *t*-test was used to assess the significance of the observed differences between control and Cd-exposed plants. In both cases, statistical significance was at *P* < 0.05.

## Results

### Effect of Short-Term Exposure to Cd under Different Sulfate Concentrations on Growth, Thiol Content, Cd Accumulation, and Sulfate Uptake of Arabidopsis Plants

**FIGURE 1:**
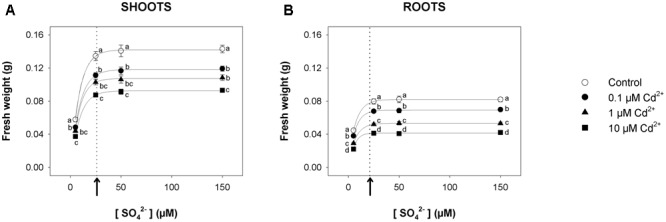
**Effect of short-term exposure to Cd on shoot and root growth as a function of the sulfate concentration in the external medium.** Arabidopsis plants were pre-grown for 19 days under four sulfate concentrations (5, 25, 50, and 150 μM) and then exposed for 72 h to three concentrations of Cd^2+^ (0.1, 1, and 10 μM). The characteristic curves describing shoot and root fresh weight accumulation in relation to the external sulfate were drawn by fitting a three-parameter exponential rise to maximum function to the data obtained by weighing the shoot and the roots of the Arabidopsis plants at the end of each experiment. **(A)** Shoot fresh weight accumulation. The correlation coefficients of the fittings were: 0.999 for the control, and 0.999, 0.999, 0.999 for plants exposed to 0.1, 1, 10 μM Cd^2+^, respectively. **(B)** Root fresh weight accumulation. The correlation coefficients of the fittings were: 0.999 for the control, and 0.998, 0.999, 0.996 for plants exposed to 0.1, 1, 10 μM Cd^2+^, respectively. Data are means and SE of two experiments run in triplicate (*n* = 6). Different letters indicate significant differences (*P* < 0.05). Dotted lines and arrows indicate [SO_4_^2–^]*_crit_*.

For short-term experiments, Arabidopsis plants were pre-grown for 19 days under four different sulfate concentrations (5, 25, 50, and 150 μM) and then exposed for 72 h to three concentrations of Cd^2+^ (0.1, 1, and 10 μM). For each Cd concentration used in the experiments, we produced a set of curves showing the dependence of shoot (**Figure 1A**) or root (**Figure 1B**) fresh weight on the sulfate availability in the growing medium. All the curves – properly described by exponential rise to maximum functions – approached saturation (95% of the maximum amount of fresh weight) at external sulfate concentrations ([SO_4_^2–^]*_crit_*) of about 26 or 21 μM, for shoots or roots, respectively. The inhibitory effect of Cd on plant growth was concentration-dependent, as indicated by the comparison of the plants grown under the same sulfate concentration. On the other hand, the effect of each Cd concentration on shoot or root fresh weight was independent of the sulfate concentration, as indicated by the analyses of the growth normalized with respect to the control (i.e., relative growth; Supplementary Figure S1).

**FIGURE 2:**
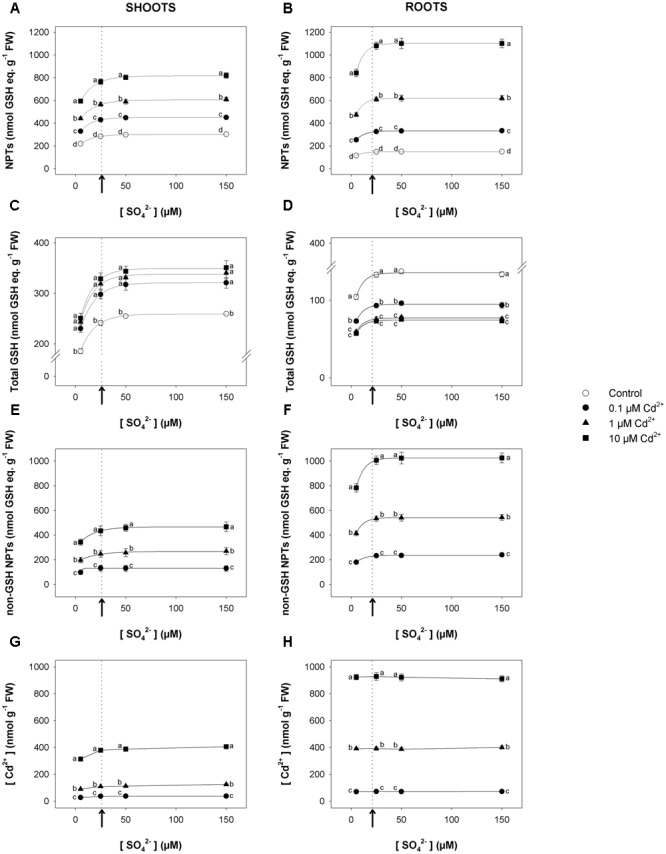
**Effect of short-term exposure to Cd on thiol and Cd levels in shoots and roots.** Arabidopsis plants were pre-grown for 19 days under four sulfate concentrations (5, 25, 50, and 150 μM) and then exposed for 72 h to three concentrations of Cd^2+^ (0.1, 1, and 10 μM). NPT levels in shoots **(A)** and roots **(B)**; total GSH levels in shoot **(C)** and roots **(D)**; PC levels in shoots **(E)** and roots **(F)**; Cd contents in shoots **(G)** and roots **(H)**. Data are means and SE of two experiments run in triplicate (*n* = 6). Different letters indicate significant differences (*P* < 0.05). Dotted lines and arrows indicate [SO_4_^2–^]*_crit_*.

Short-term exposure to Cd significantly changed the NPT levels of both shoots and roots, which increased as Cd concentration did under all the sulfate concentrations analyzed (**Figures 2A,B**). Such trends were mainly related to the accumulation of non-GSH NPTs (mainly PCs and related peptides), which became the most abundant class of thiols in the tissues of Cd-exposed plants (**Figures 2E,F**). Opposite behaviors were observed as regards the effects of Cd on the total GSH levels of shoots and roots. In fact, for each sulfate concentration, the levels of total GSH significantly increased or decreased, considering shoots or roots, respectively, as the Cd concentration in the growing medium increased (**Figures 2C,D**). Moreover, for each Cd concentration, the dependence of the NPT, total GSH, and non-GSH NPT levels on the external sulfate was described by typical exponential rise to maximum curves, approaching the saturation at sulfate concentrations very close to the critical ones. Finally, Cd concentration was higher in the roots than in the shoots; its concentration in the shoot or root tissues was dependent on the level of the metal in the growing medium, but was unaffected by the sulfate concentration (**Figures 2G,H**).

**FIGURE 3:**
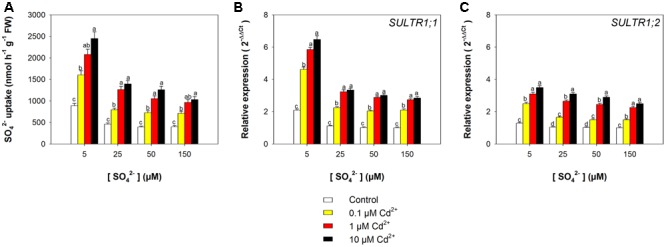
**Effect of short-term exposure to Cd on the sulfate uptake capacity of the roots.** Arabidopsis plants were pre-grown for 19 days under four sulfate concentrations (5, 25, 50, and 150 μM) and then exposed for 72 h to three concentrations of Cd^2+^ (0.1, 1, and 10 μM). **(A)** Sulfate uptake capacity was evaluated by measuring the rate of ^35^SO_4_^2–^ absorption into roots of intact plants over a 15 min pulse. The incubation solutions contained 150 μM SO_4_^2–^. **(B,C)** Changes in the relative transcript levels of *SULTR1; 1* and *SULTR1; 2* in the roots. Bars and error bars are means and SE of two experiments run in triplicate (*n* = 6). Different letters indicate significant differences (*P* < 0.05).

The capacity of the Arabidopsis roots to take up sulfate was strongly affected by the sulfate availability in the growing medium, as well as by the presence of Cd, as indicated by the values of ^35^S-sulfate uptake measured at 150 μM SO_4_^2–^ external concentration (**Figure 3A**). In the control plants, the rate of sulfate uptake increased up to 1.2-fold, moving the external sulfate concentration from 150 to 5 μM. A Cd-dependent increase in the rate of sulfate uptake was also observed in the Arabidopsis plants grown under the same sulfate concentration in the media. Sulfate uptake increases of 1.7-, 2.0-, 2.2-, and 1.6-fold were measured in plants grown under 5, 25, 50, and 150 μM external sulfate, respectively, moving the Cd^2+^ concentration from 0 to 10 μM. These behaviors were closely associated to changes in the relative transcript levels of *SULTR1; 1* (**Figure 3B**) and *SULTR1; 2* (**Figure 3C**), the two Arabidopsis genes involved in sulfate uptake by roots (Maruyama-Nakashita et al., 2004).

### Effect of Long-Term Exposures to Cd under Different Sulfate Concentrations on Growth, Thiol Content, Cd Accumulation, and Sulfate Uptake of Arabidopsis Plants

**FIGURE 4:**
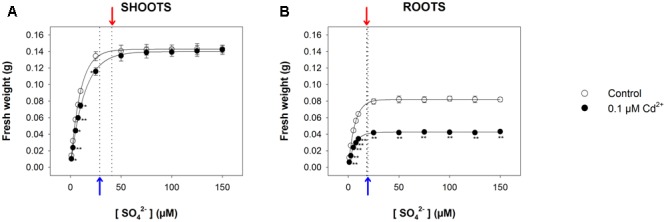
**Effect of long-term exposure to Cd on shoot and root growth as a function of the sulfate concentration in the external medium.** Arabidopsis plants were grown for 22 days under a wide range of sulfate concentrations (1, 2.5, 5, 7.5, 10, 25, 50, 75, 100, 125, and 150 μM) in the presence or absence of 0.1 μM Cd^2+^. The characteristic curves describing shoot and root fresh weight accumulation in relation to the external sulfate were drawn by fitting a three-parameter exponential rise to maximum function to the data obtained by weighing the shoot and the roots of the Arabidopsis plants at the end of each experiment. **(A)** Shoot fresh weight accumulation. The correlation coefficients of the fittings were 0.999 and 0.999, for control and Cd-exposed plants, respectively. **(B)** Root fresh weight accumulation. The correlation coefficients of the fittings were 0.999 and 0.999, for control and Cd-exposed plants, respectively. Data are means and SE of two experiments run in triplicate (*n* = 6). Asterisks indicate significant differences (Student’s *t*-test; ^∗^0.001 ≤*P* < 0.05; ^∗∗^*P* < 0.001) between control and Cd-exposed plants grown under the same sulfate external concentration. Blue and red arrows indicate the values of [SO_4_^2–^]*_crit_* calculated for control and Cd-exposed plants, respectively.

For long-term exposure experiments, we grew Arabidopsis plants for 22 days under a wide range of sulfate concentrations (1, 2.5, 5, 7.5, 10, 25, 50, 75, 100, 125, and 150 μM MgSO_4_), in the presence or absence of 0.1 μM CdCl_2_. As previously shown, the data set distribution of shoot and root fresh weights (obtained in each treatment condition) as a function of the external sulfate concentration was properly described by an exponential rise to maximum function, which allowed us to calculate the relative [SO_4_^2–^]*_crit_* maximizing the growth in each experimental condition. Such a value, calculated for the shoots (**Figure 4A**), was significantly higher in Cd-treated (40.9 ± 1.2 μM) than in control plants (28.8 ± 0.6 μM), whilst, for the roots (**Figure 4B**), was independent of the presence of Cd (19.4 ± 0.3 μM and 18.3 ± 0.5 μM, for control and Cd-treated plants, respectively). Interestingly, the analysis of the growth curves also revealed that Cd exposure exerted inhibitory effects on shoot growth only at sulfate concentrations lower than [SO_4_^2–^]*_crit_*. At sulfate external concentrations higher than [SO_4_^2–^]*_crit_* the presence of Cd did not affect shoot growth (**Figure 4A**). Moreover, the effects produced by Cd on shoot growth were closely dependent on the sulfate concentration, since they reduced as the sulfate concentration in the medium increased up to the value of [SO_4_^2–^]*_crit_* (Supplementary Figure S2A). Conversely, the effects of Cd on root growth were independent of the sulfate concentration in the growing medium (Supplementary Figure S2B).

**FIGURE 5:**
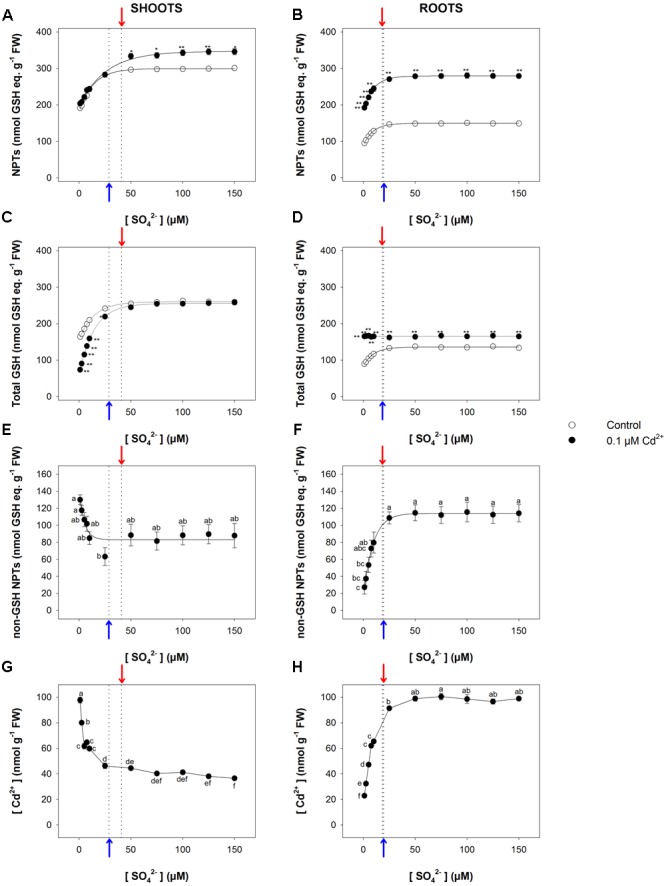
**Effect of long-term exposure to Cd on thiol and Cd levels in shoots and roots.** Arabidopsis plants were grown for 22 days under a wide range of sulfate concentrations (1, 2.5, 5, 7.5, 10, 25, 50, 75, 100, 125, and 150 μM) in the presence or absence of 0.1 μM Cd^2+^. NPT levels in shoots **(A)** and roots **(B)**; total GSH levels in shoot **(C)** and roots **(D)**; PC levels in shoots **(E)** and roots **(F)**; Cd contents in shoots **(G)** and roots **(H)**. Data are means and SE of two experiments run in triplicate (*n* = 6). Different letters indicate significant differences (*P* < 0.05). Asterisks indicate significant differences (Student’s *t*-test; ^∗^0.001 ≤*P* < 0.05; ^∗∗^*P* < 0.001) between control and Cd-exposed plants grown under the same sulfate external concentration. Blue and red arrows indicate the values of [SO_4_^2–^]*_crit_* calculated for control and Cd-exposed plants, respectively.

The relationship between the NPT level and the sulfate concentration in the medium exhibited a saturation behavior for both shoots and roots (**Figures 5A,B**). In particular, the NPT levels measured in the shoots were significantly higher in Cd-exposed than in control plants at sulfate external concentrations higher than [SO_4_^2–^]*_crit_*; no significant effects were observed at sulfate concentrations lower than [SO_4_^2–^]*_crit_* (**Figure 5A**). On the other hand, the NPT levels in the Cd-exposed roots were significantly higher than in the control under all the sulfate concentrations analyzed (**Figure 5B**). Such behaviors were associated with notable changes in the balance among the different classes of thiols, whose relative abundance seemed to be dependent on: (i) the presence/absence of Cd in the growing medium; (ii) the sulfate external concentration; (iii) the plant tissues we considered. The analyses of the curves describing the dependence of the total GSH levels of the shoots on the sulfate external concentration revealed that the inhibitory effects exerted by Cd on the GSH accumulation gradually decreased, moving the external sulfate concentration up to the critical value calculated for this condition. At sulfate external concentrations higher than [SO_4_^2–^]*_crit_* the presence of Cd did not affect the total GSH levels, whose values were similar to those measured in the control (**Figure 5C**). Such behavior was closely related to the levels of the non-GSH NPT in the shoot tissues, which progressively decreased as the sulfate concentration increased, up to a constant value at sulfate concentrations higher than [SO_4_^2–^]*_crit_* (**Figure 5E**). A different picture was evinced by analyzing the dependence of total GSH and non-GSH NPT levels of the roots on the external sulfate concentration. Interestingly, in Cd-exposed roots the total GSH levels were significantly higher than in the control and did not show any apparent dependence on the sulfate external concentration (**Figure 5D**), differently from the non-GSH NPT levels, whose dependence on the sulfate external concentration was properly described by a saturation curve (**Figure 5F**). Finally, it is worth noting that the concentration of Cd in both shoots and roots was not constant under all the sulfate concentration analyzed, showing a dependence on sulfate external concentration similar to that described for the concentrations of the non-GSH NPTs in each apparatus (**Figures 5G,H**).

**FIGURE 6:**
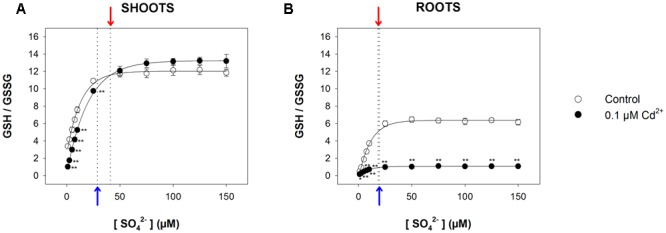
**Effect of long-term exposure to Cd on the GSH/GSSG ratio in shoots (A)** and roots **(B)**. Arabidopsis plants were grown for 22 days under a wide range of sulfate concentrations (1, 2.5, 5, 7.5, 10, 25, 50, 75, 100, 125, and 150 μM) in the presence or absence of 0.1 μM Cd^2+^. The GSH/GSSG ratios were calculated using data about reduced GSH and GSSG reported in Supplementary Figure S3. Data are means and SE of two experiments run in triplicate (*n* = 6). Asterisks indicate significant differences (Student’s *t*-test; ^∗^0.001 ≤ *P* < 0.05; ^∗∗^*P* < 0.001) between control and Cd-exposed plants grown under the same sulfate external concentration. Blue and red arrows indicate the values of [SO_4_^2–^]_crit_ calculated for control and Cd-exposed plants, respectively.

Since reduced GSH not only represents the key intermediate for the synthesis of PCs, but also plays a pivotal role as an antioxidant in controlling the cellular redox status, we measured the GSH/GSSG ratio in all the experimental conditions, assuming this value as a marker for oxidative stress. Results (**Figure 6** and Supplementary Figure S3) revealed that in this case also, the relationship between the GSH/GSSG ratio measured in both shoots and roots and the sulfate concentration in the medium was described, in each condition, by a saturation curve, indicating that the optimal cellular redox status was reached at sulfate concentrations higher than the respective critical values. In particular, the GSH/GSSG ratio measured in the shoots was significantly lower in Cd-exposed than in control plants at sulfate external concentrations lower than [SO_4_^2–^]*_crit_*; no significant effects were observed at sulfate concentrations higher than [SO_4_^2–^]*_crit_* (**Figure 6A**). Conversely, the GSH/GSSG ratio in the Cd-exposed roots were significantly lower than in the control under all the sulfate concentrations analyzed (**Figure 6B**).

**FIGURE 7:**
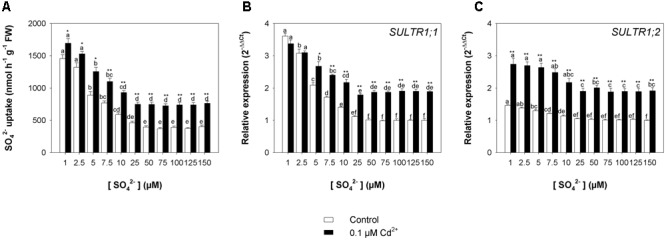
**Effect of long-term exposure to Cd on the sulfate uptake capacity of the roots.** Arabidopsis plants were grown for 22 days under a wide range of sulfate concentrations (1, 2.5, 5, 7.5, 10, 25, 50, 75, 100, 125, and 150 μM) in the presence or absence of 0.1 μM Cd^2+^. **(A)** Sulfate uptake capacity was evaluated by measuring the rate of ^35^SO_4_^2–^ absorption into roots of intact plants over a 15-min pulse. The incubation solutions contained 150 μM SO_4_^2–^. **(B,C)** Changes in the relative transcript levels of *SULTR1; 1* and *SULTR1; 2* in the roots. Bars and error bars are means and SE of two experiments run in triplicate (*n* = 6). Different letters indicate significant differences (*P* < 0.05). Asterisks indicate significant differences (Student’s *t*-test; ^∗^0.001 ≤ *P* < 0.05; ^∗∗^*P* < 0.001) between control and Cd-exposed plants grown under the same sulfate external concentration.

Finally, the rate of sulfate uptake – measured at 150 μM SO_4_^2–^ external concentration at the end of the exposure period – increased as the sulfate concentration in the growing medium decreased, but was significantly higher in Cd-exposed than in control plants under all the sulfate concentrations analyzed (**Figure 7A**). Close relationships between the rate of sulfate uptake and the relative transcript levels of *SULTR1; 1* (**Figure 7B**) and *SULTR1; 2* (**Figure 7C**) were also observed in this set of experiments.

## Discussion

Several papers report that early Cd stress triggers a wide range of adaptive mechanisms – involving GSH consuming activities – which may increase the metabolic demand for sulfate, sulfur metabolites and carbon skeletons (Lee and Leustek, 1999; Nocito et al., 2006, 2008). In fact GSH not only is polymerized to form PCs in response to Cd accumulation (Xiang et al., 2001; Rea et al., 2004), but also acts as an antioxidant in mitigating the oxidative stress produced by free Cd^2+^ ions into the cells (Cuypers et al., 2011; Noctor et al., 2012). In such a context, the need to maintain GSH homeostasis and continuous Cd chelation induces responses allowing plants to increase sulfate uptake by roots and sulfate entry in the reductive assimilation pathway, as well as to modulate sulfate allocation among the different tissues and organs. Such responses are mainly controlled at transcriptional level and involve transcript accumulation of genes that encode sulfate transporters and activities along the pathways of sulfate assimilation and GSH biosynthesis (Lee and Leustek, 1999; Nocito et al., 2002, 2006; Lancilli et al., 2014; Yamaguchi et al., 2016). The pivotal importance of sulfate uptake in the plant adaptation to Cd stress has recently been underlined by the analysis of the Arabidopsis *sultr1; 1-sultr1; 2* double mutant – defective in two distinct high-affinity sulfate transporters (SULTR1; 1 and SULTR1; 2) involved in root sulfate uptake from the rhizosphere – which resulted, under limited sulfate supply, in plants being more sensitive to Cd-induced oxidative stress than the wild type (Liu et al., 2016). Moreover, analyses of Arabidopsis mutants defective in thiol metabolism and accumulation revealed that both oxidative stress and thiol depletion are necessary to induce the transcription of *SULTR1; 2* during early Cd stress (Jobe et al., 2012). Thus, while the contribution of Cd-induced sulfate uptake to the early phases of Cd-detoxification appears evident, the role of sulfate uptake and S nutrition in maintaining plant growth under prolonged Cd exposure still needs to be elucidated, since long-term exposure to Cd may permanently affect thiol homeostasis and allocation to different sinks, ultimately affecting the plant’s capacity to optimize its growth at a given sulfate concentration in the external medium.

The analyses of the dependence of both shoots and root fresh weight on the amount of external sulfate available for growth (**Figures 1, 4**) may allow some educated guesses on how plants optimize growth at a given sulfate concentration. For this reason we calculated for each condition the [SO_4_^2–^]*_crit_* (i.e., the sulfate concentration in the growing medium that produced 95% of the maximum amount of fresh weight for shoots or roots), assuming that changes in this value necessarily reflect changes in the plant’s ability to use the external S sources to promote growth.

Starting from the deficiency range, increasing the sulfate supply increases plant growth although the response diminishes as the supply of sulfate ions is increased according to the low of diminishing yield increment formulated by McNall (1933), Mitscherlich (1954), and Engels et al. (2012). In the second part of the curves (i.e., at sulfate concentration higher than [SO_4_^2–^]*_crit_*) increasing of the sulfate supply does not produce any significant growth increment, indicating that other nutrients, environmental factors or the “genetic potential” of the plant become limiting factors.

Data analysis reveals that short-term exposure to Cd negatively affects plant growth but does not produce any significant effects on the growth pattern of shoots or roots in relation to the external sulfate, as indicated by the invariance of the [SO_4_^2–^]*_crit_* determined for shoot or root growth under each experimental condition (**Figure 1** and Supplementary Figure S1). On the other hand, long-term exposure to Cd significantly changes the pattern of fresh weight accumulation of the shoots in relation to the external sulfate, and significantly enhances the [SO_4_^2–^]*_crit_* maximizing the growth (**Figure 4A**). It is also worth noting that in this condition, increasing the sulfate external concentration up to the [SO_4_^2–^]*_crit_* progressively reduces the inhibitory effects exerted by the same concentration of Cd on shoot growth (Supplementary Figure S2A), indicating that – at least in our conditions – plant tolerance to relatively low Cd concentrations is dependent to the S nutritional status, as previously observed in maize seedlings grown under different sulfate availabilities (Nocito et al., 2006). Such a behavior seems to be related to multiple and complex effects induced by the increase of the sulfate concentration in a range of sub-optimal availability for plant growth, which by affecting thiol biosynthesis produces serious effects on non-GSH NPT accumulation, Cd partitioning between shoots and roots, and cellular redox state (**Figures 5, 6**). In such a scenario, the increase in the non-GSH NPT levels of the roots (mainly PCs and related peptides) induced by enhancing sulfate (**Figure 5F**) progressively results in a greater capacity to retain Cd within the roots (**Figure 5H**), and thus reduces the amount of free Cd^2+^ ions that – escaping chelation – is potentially available to be translocated via the xylem in a root-to-shoot direction (Wong and Cobbett, 2009; Nocito et al., 2011). The analysis of changes in the shoot Cd concentration in relation to the external sulfate (**Figure 5G**) further supports this conclusion, underlining that the sulfate-induced enhancement in Cd root retention contributes to reduce Cd accumulation and injury in the shoot tissues (**Figures 4A, 5G**, and Supplementary Figure S2A). Moreover, the increase in the sulfate external concentration progressively reduces the negative effect of Cd on the level of reduced GSH in the shoots (Supplementary Figure S3A), enhancing the cellular capacity to cope with Cd-induced oxidative stress. Such an effect allows the shoot tissues to progressively contrast the oxidative damage exerted by Cd, until reaching complete recovery at sulfate concentrations higher than [SO_4_^2–^]*_crit_*, i.e., where the cells of the shoot tissues reached the optimal redox status, as indicated by the values of GSH/GSSG ratio that we assume as indicators of oxidative stress (**Figure 6A**). Conversely, the lack in the roots of a “sulfate-induced recovery” from Cd damage (**Figure 4B** and Supplementary Figure S2B) indicates that the increase in the total GSH levels of the roots induced by long-term exposure to Cd may be not enough to fully sustain Cd detoxification processes and thus to efficiently counteract the redox imbalance produced by Cd in the root tissues (**Figures 5D, 6B**).

Taken as a whole the behaviors discussed so far clearly suggest that the sulfate-dependent adaptive responses to long-term exposure to Cd may be part of the general physiological and biochemical mechanisms acting as a “firewall” to prevent excessive Cd accumulation in the shoot tissues (Mendoza-Cózatl et al., 2011). The efficiency of these responses seems to be related to the concentration of the sulfate ions in the growing medium, since the shoot recovery from Cd stress requires a higher sulfate concentration than that required to maximize shoot growth in the absence of Cd.

**FIGURE 8:**
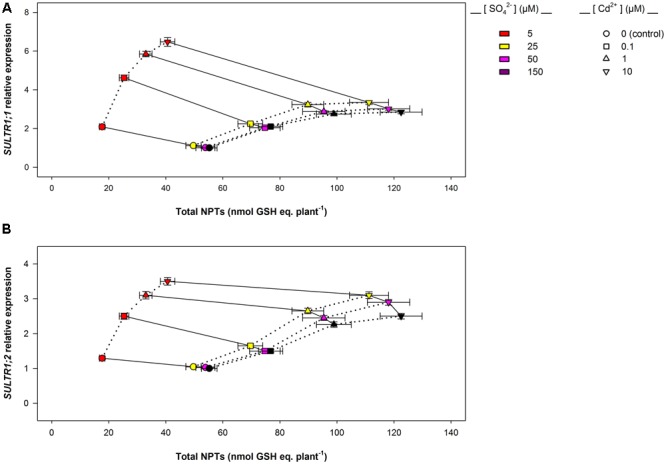
***SULTR1; 1* (A)** and *SULTR1; 2* **(B)** relative expression vs. total NPT levels per plant: short-term exposure. The relationships between the relative expression of *SULTR1; 1* or *SULTR1; 2* transcript and the total amount of NPTs per plant were evinced using data reported in **Figure 3** and Supplementary Figure S4. Solid lines link data about plants exposed to the same Cd concentration (circles, 0 μM Cd^2+^; squares, 0.1 μM Cd^2+^; triangles up, 1 μM Cd^2+^; triangles down, 10 μM Cd^2+^), under different sulfate availabilities. Dotted lines link data about plants grown under the same sulfate concentration in the absence or presence of different Cd^2+^ concentrations. Data reported in each plot are means and SE of two experiments run in triplicate (*n* = 6).

**FIGURE 9:**
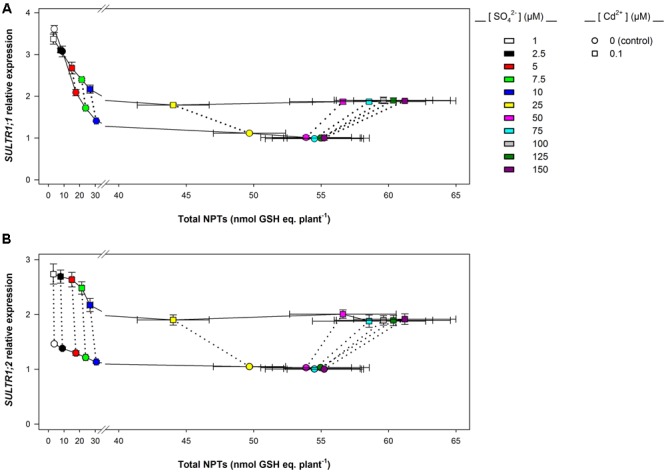
***SULTR1; 1* (A)** and *SULTR1; 2* **(B)** relative expression vs. total NPT levels per plant: long-term exposure. The relationships between the relative expression of *SULTR1; 1* or *SULTR1; 2* transcript and the total amount of NPTs per plant were evinced using data reported in **Figure 7** and Supplementary Figure S4. Solid lines link data about plants grown under different sulfate concentrations in the absence (circles) or presence of 0.1 μM Cd^2+^ (squares). Dotted lines link data about plants grown under the same sulfate concentration. Data reported in each plot are means and SE of two experiments run in triplicate (*n* = 6).

Data analysis also reveals that the induction of sulfate uptake is a common adaptive response to both short- and long-term exposure to Cd (**Figures 3A, 7A**). In fact, under all the sulfate concentrations analyzed, the presence of Cd in the growing medium modulates the well-known effects of the sulfate external concentration on the capacity of the Arabidopsis roots to take up sulfate (Takahashi et al., 1997; Shibagaki et al., 2002; Yoshimoto et al., 2002, 2007; Rouached et al., 2008). Such a modulation seems to be related to a differential regulation of the transcription of *SULTR1; 1* and *SULTR1; 2*, the two Arabidopsis genes involved in sulfate uptake by roots (**Figures 3B,C, 7B,C**), probably as a consequence of the Cd-induced changes in thiol metabolism and partitioning. However, a careful comparison of the amounts of the NPTs accumulated in each plant, in the presence or absence of Cd, clearly reveals the existence of a differential and time-dependent effect of Cd exposure on thiol accumulation, and thus on the nutritional need for S generated by Cd (Supplementary Figure S4). In our experiments, short-term exposure to Cd decreases the level of total S accumulated by a single plant (Supplementary Figure S4C) – probably in relation to the negative effect of the metal on shoot and root growth – and induces additional sinks for thiols whose strengths are closely dependent on the concentration of the metal in the growing medium (Supplementary Figure S4A), as previously reported by Lancilli et al. (2014). In such conditions, the increase in the relative expression of *SULT1; 1* and *SULTR1; 2* seems to be due to homeostatic mechanisms driven by the Cd-induced increase in the total NPT levels per plant, since under all the sulfate concentrations analyzed, the presence of 0.1, 1, or 10 μM Cd^2+^ positively affected both sulfate transporter gene expression and total NPT levels (**Figures 8A,B**). Conversely, long term-exposure to Cd does not produce additional sinks for thiols and significantly reduces the level of total S accumulated by each plant grown under sulfate external concentration lower than [SO_4_^2–^]*_crit_* (Supplementary Figures S4B,D). In this condition the total amount of NPTs per plant, calculated for each sulfate concentration, was not significantly affected by the presence of 0.1 μM Cd^2+^ (Supplementary Figure S4B), and the relationship between changes in the relative expression of *SULTR1; 1* or *SULTR1; 2* transcript and the total amount of NPTs per plant appears to be more complex than those described under short-term exposure to Cd (**Figures 9A,B**). Considering the plots in **Figures 9A,B** we can easily evince that, under the same sulfate concentration, the Cd-induced increase in transcript level of *SULTR1; 1* or *SULTR1; 2* does not produce any significant changes in the total amount of NPTs per plant. Such a finding indicates that long-term exposure to Cd, even if results in a greater rate of sulfate uptake (**Figure 7A**), negatively affects the capacity of the entire root apparatus to absorb the external sulfate at concentration lower than [SO_4_^2–^]*_crit_*, probably because of the dramatic effect produced by Cd on the pattern of fresh weight accumulation of the roots (**Figure 4B**). Such a behavior clearly indicates that the interaction between the molecular mechanisms controlling sulfate uptake and root development under different sulfate concentrations may limit the capacity of the root apparatus to optimize the external sulfate supply under long-term exposure to Cd. The capacity of the roots to adequate sulfate fluxes and sulfate transporter gene expression in the sulfate deficiency range (**Figure 7**) indicates that below the [SO_4_^2–^]*_crit_* – calculated for the shoots of the control plants – both sulfate supply and relative expressions of SULTR1; 1 and SULTR1; 2 limit the Arabidopsis growth potential in our environmental conditions (i.e., light, photoperiod, temperature, nutrient availability, etc.). The presence of Cd in the growing medium significantly alters thiol metabolism and partitioning, promotes sulfate uptake and increases the [SO_4_^2–^]*_crit_*, but does not change the range of sulfate external concentrations in which sulfate uptake appears to be modulated (e.g., from 1 to 25 μM), suggesting that sulfate perception in the growing medium may play an important role in this response. Recent advances in our knowledge of the mechanisms controlling sulfate uptake and assimilation point out that these pathways are not only regulated by the demand for reduced sulfur, but also by phytohormones and various environmental factors, such as extracellular sulfate availability (Koprivova and Kopriva, 2014; Zheng et al., 2014). Physiological evidences have shown that the sulfate transporter SULTR1; 2 of Arabidopsis may has an additional role in the extracellular S sensing that could be independent of the perception of the internal S status or nutritional request, since mutations in the relative gene reduce sensitivity to the S-transcriptional repression of key S-responsive genes (Zhang et al., 2014). In this context, sensing of extracellular sulfate may limit further sulfate uptake under long-term exposure to Cd, even if the metabolic demand for reduced sulfur is high, and thus reduces the capacity of the plants to optimize sulfate uptake in relation to root growth. In fact, under long term-exposure to Cd the relative growth of the roots was only about 52.5% with respect to the control, under all the sulfate concentrations analyzed, whilst the -fold change for the potential capacity of the roots to take up sulfate (measured at saturation) ranged from 0.2 to 1, moving the sulfate external concentration from 1 to 150 μM. From these data we can calculate that Cd-exposed plants became able to absorb the same amount of sulfate as control plants at 56.9 μM external SO_4_^2–^, i.e., where the -fold change for the potential capacity of the roots to take-up sulfate reached the value of 0.9 (Supplementary Figure S5). In these conditions ([SO_4_^2–^]*_out_* ≥ 56.9 μM), the induction of sulfate uptake is potentially able to balance the negative effects of Cd on root growth and then to assure an adequate sulfate amount for optimizing shoot growth and thiol metabolism.

## Conclusion

Our results indicate that long term-exposure to Cd, although it induces sulfate uptake, decreases the capacity of the Arabidopsis roots to efficiently absorb the sulfate ions available in the growing medium to promote growth. Such a behavior is likely due to an effect exerted by Cd accumulation which – by reducing the development of the root apparatus – makes the adaptive response of the high-affinity sulfate transporters “*per se*” not enough to optimize the growth at sulfate external concentrations lower than [SO_4_^2–^]*_crit_*.

## Author Contributions

FN, CL, and GAS conceived and designed the experiments. AF, MM, and FN performed the experiments and analyzed the data. GL performed the ICP-MS analysis. GAS acquired the funds. FN and AF wrote the manuscript. All authors discussed, revised, and approved the manuscript.

## Conflict of Interest Statement

The authors declare that the research was conducted in the absence of any commercial or financial relationships that could be construed as a potential conflict of interest.
