# Supplementary material for: The Sulfate Supply Maximizing Arabidopsis Shoot Growth Is Higher under Long- than Short-Term Exposure to Cadmium
Source: Front Plant Sci. 2017 May 22;8:854. doi: 10.3389/fpls.2017.00854 (PMC5439006; doi:10.3389/fpls.2017.00854)
Supplement: Supplementary file 1 [file Data_Sheet_1.pdf]

Supplementary Material

The sulfate supply maximizing Arabidopsis shoot growth is higher under long- than short-term exposure to cadmium

Alessandro Ferri, Clarissa Lancilli, Moez Maghrebi, Giorgio Lucchini, Gian Attilio Sacchi, Fabio Francesco Nocito*

*** Correspondence:** Fabio Francesco Nocito: fabio.nocito@unimi.it

Supplementary Table S1. Primers used for qRT-PCR analysis.

Gene	Primer name	Sequence
SULTR1;1 (At4g08620)	Sultr1;1F	GCCATCACAATCGCTCTCCAA
	Sultr1;1R	TTGCCAATTCCACCCATGC
SULTR1;2 (At1g78000)	Sultr1;2F	GGATCCAGAGATGGCTACATGA
	Sultr1;2R	TCGATGTCCGTAACAGGTGAC
S16 (At4g34620)	S16F	CGCCGATCGAGCTTTATCAG
	S16R	CACCAGGACCACCAAACTTCTT

Supplementary Figure S1

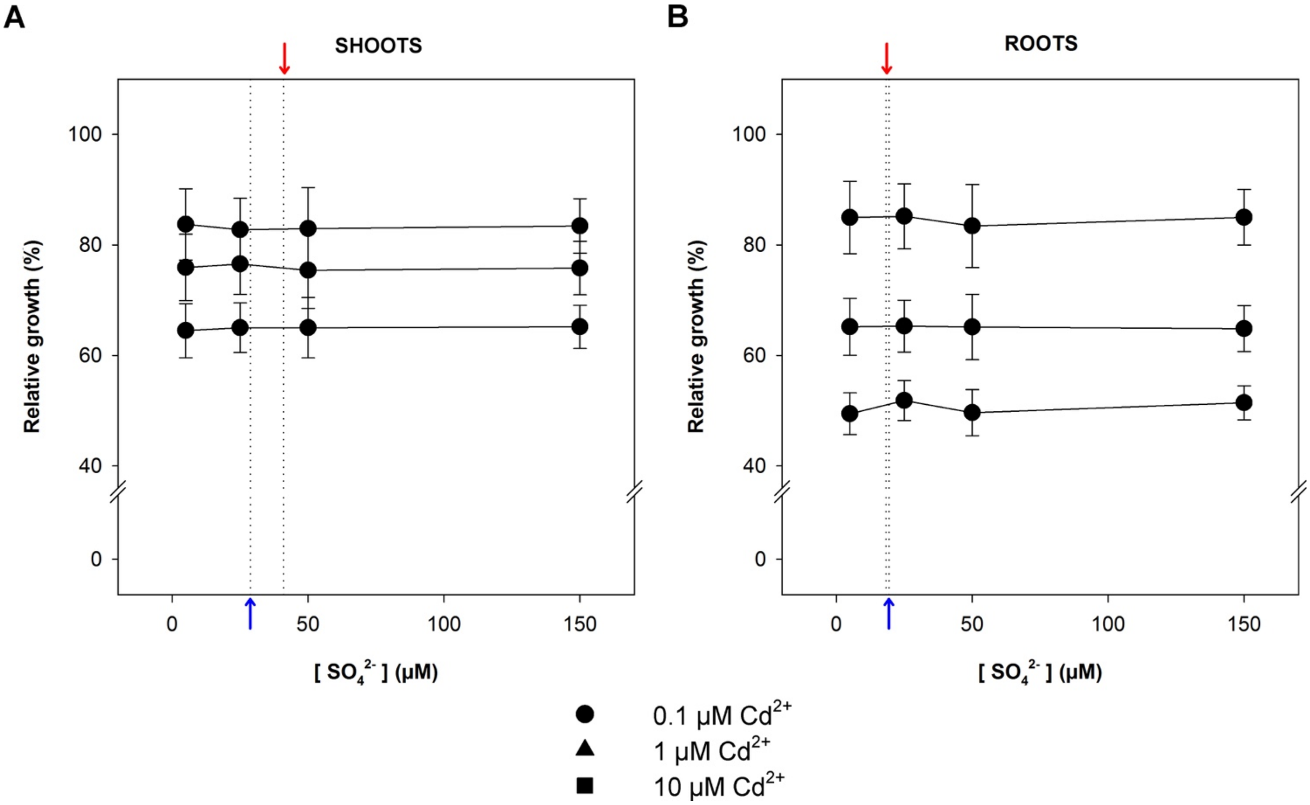

Shoot (A) and root (B) relative growth under short-term exposure to Cd. Arabidopsis plants were pre-grown for 19 days under four sulfate concentrations (5, 25, 50 and 150 μM) and then exposed for 72 h to three concentrations of Cd^{2+} (0.1, 1 and 10 μM). Relative growths for shoots and roots were calculated using data reported in Figure 1, by normalizing the growth of Cd-exposed plants with respect to the control. Data are means and SE of two experiments run in triplicate ($n = 6$). Blue and red arrows indicate the values of $[SO_4^{2-}]_{crit}$ calculated for control and Cd-exposed plants, respectively.

Supplementary Figure S2

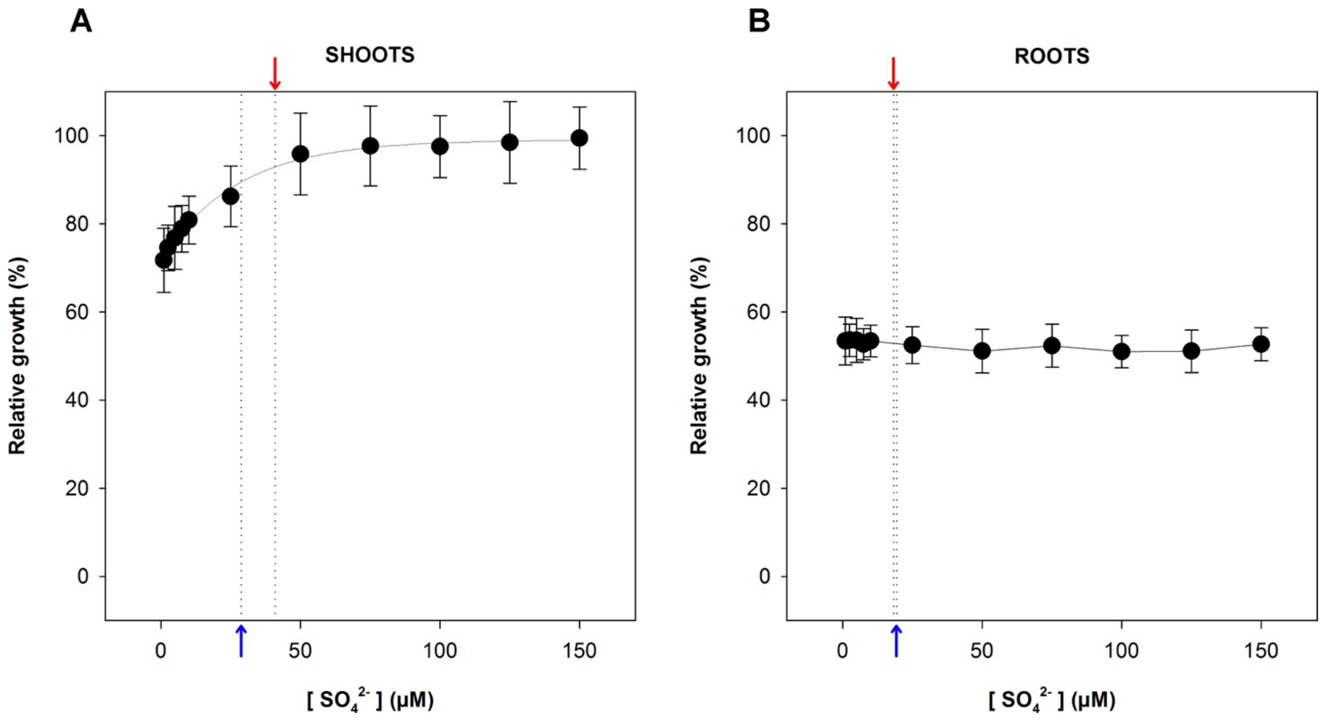

Shoot (A) and root (B) relative growth under long-term exposure to Cd. Arabidopsis plants were grown for 22 days under a wide range of sulfate concentrations (1, 2.5, 5, 7.5, 10, 25, 50, 75, 100, 125 and 150 µM) in the presence or absence of 0.1 µM Cd^{2+}. Relative growths for shoots and roots were calculated using data reported in Figure 4, by normalizing the growth of Cd-exposed plants with respect to the control. Data are means and SE of two experiments run in triplicate (*n* = 6). Blue and red arrows indicate the values of $[SO_4^{2-}]_{crit}$ calculated for control and Cd-exposed plants, respectively.

Supplementary Figure S3

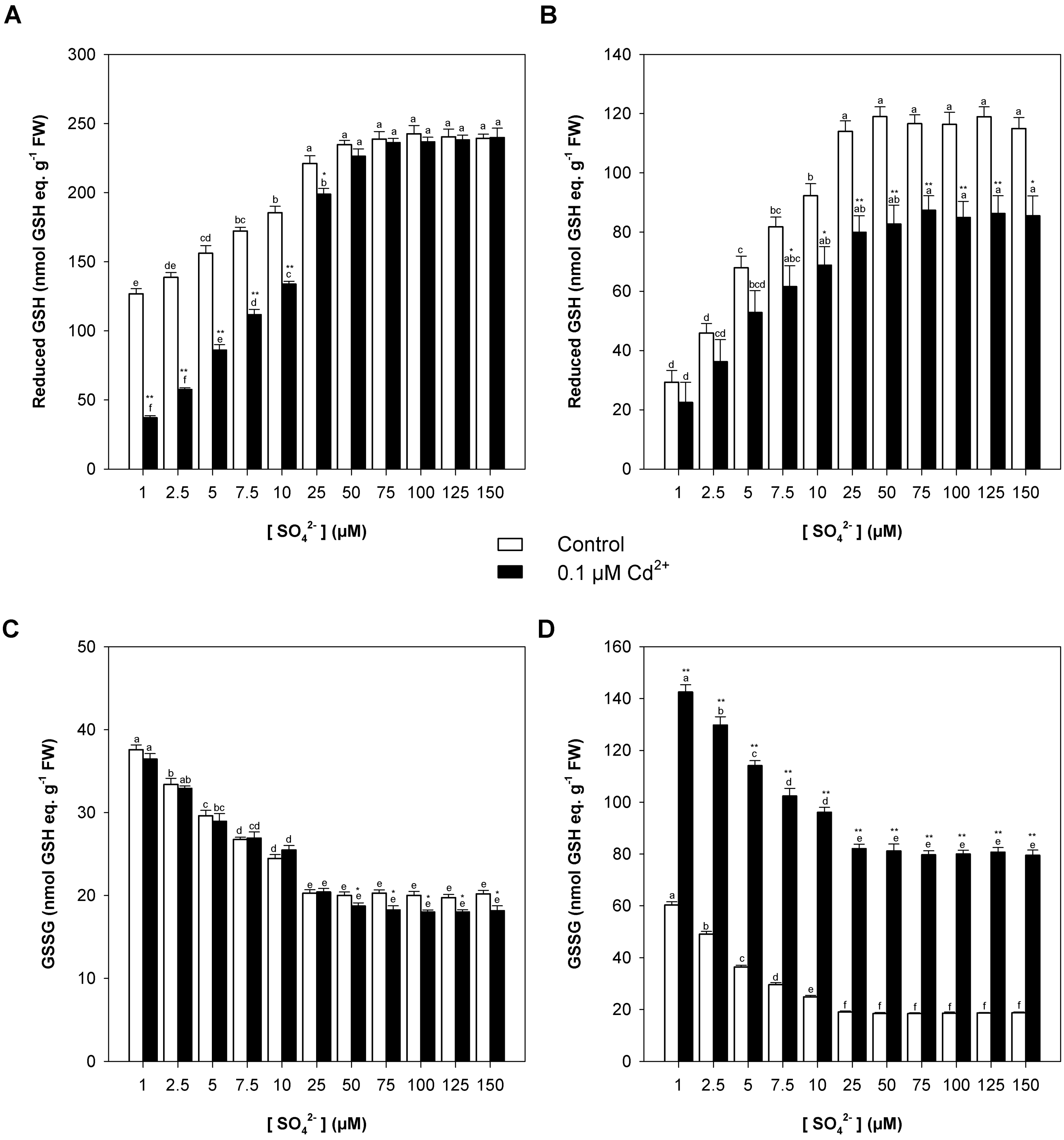

Effect of long-term exposure to Cd on reduced GSH and GSSG levels in shoots and roots. Arabidopsis plants were grown for 22 days under a wide range of sulfate concentrations (1, 2.5, 5, 7.5, 10, 25, 50, 75, 100, 125 and 150 µM) in the presence or absence of 0.1 µM Cd^{2+}. Reduced GSH levels in shoots **(A)** and roots **(B)**; GSSG levels in shoots **(C)** and roots **(D)**. Bars and error bars are means and SE of two experiments run in triplicate ($n = 6$). Different letters indicate significant differences ($P < 0.05$). Asterisks indicate significant differences (Student's *t*-test; * $0.001 \leq P < 0.05$; ** $P < 0.001$) between control and Cd-exposed plants grown under the same sulfate external concentration.

Supplementary Figure S4

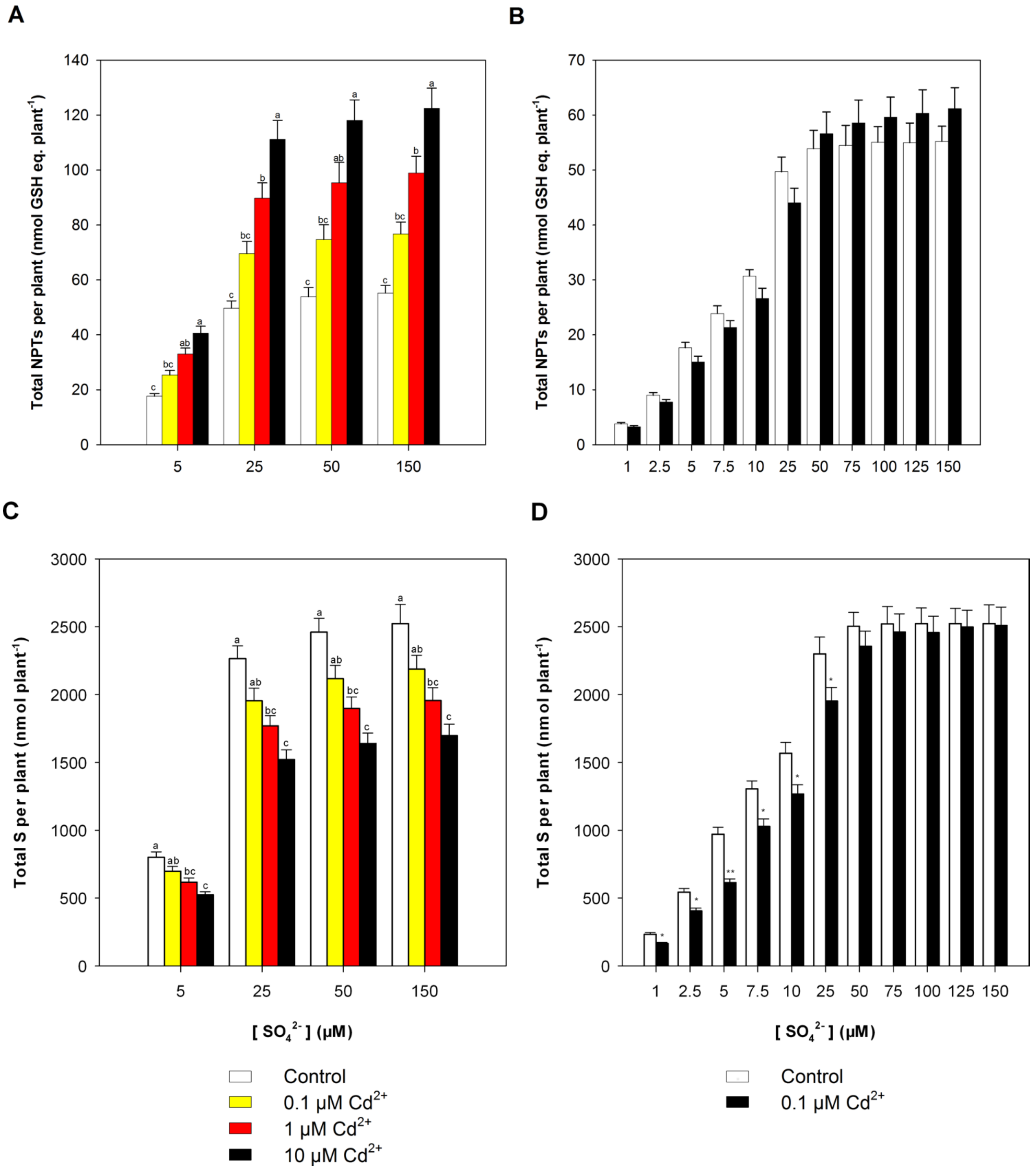

Effect of short- and long-term exposure to Cd on the total NPT (A,B) and the total S (C,D) levels per plant. For the short-term exposure to Cd, Arabidopsis plants were pre-grown for 19 days under four sulfate concentrations (5, 25, 50 and 150 μM) and then exposed for 72 h to three concentrations of Cd^{2+} (0.1, 1 and 10 μM). For the long-term exposure to Cd, Arabidopsis plants were grown for 22 days under a wide range of sulfate concentrations (1, 2.5, 5, 7.5, 10, 25, 50, 75, 100, 125 and 150 μM) in the presence or absence of 0.1 μM Cd^{2+}. The total NPT levels per plant were calculated using data reported in Figures 1, 2(A,B), 4, 5(A,B). Bars and error bars are means and SE of two experiments run in triplicate ($n = 6$). Different letters indicate significant differences ($P < 0.05$). Asterisks indicate significant differences (Student's t-test; * $0.001 \leq P < 0.05$; ** $P < 0.001$) between control and Cd-exposed plants grown under the same sulfate external concentration.

Supplementary Figure S5

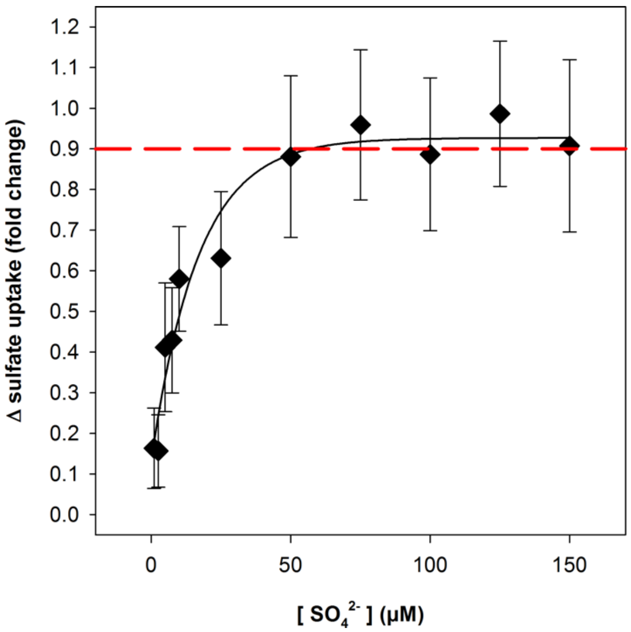

Cd-induced changes in the potential capacity of the roots to take up sulfate as a function of the sulfate external concentration. The analysis was performed using data reported in Figure 7A. The red line indicates the threshold over which sulfate uptake is potentially able to balance the negative effects of long-term exposure to Cd on root growth and then to assure an adequate sulfate amount for optimizing shoot growth and thiol metabolism. Data reported in each plot are means and SE of two experiments run in triplicate ($n = 6$).
